# Myelin-specific T helper 17 cells promote adult hippocampal neurogenesis through indirect mechanisms

**DOI:** 10.12688/f1000research.4439.2

**Published:** 2017-04-07

**Authors:** Johannes Niebling, Annette E. Rünker, Sonja Schallenberg, Karsten Kretschmer, Gerd Kempermann

**Affiliations:** 1 Molecular and Cellular Immunology/Immune Regulation, CRTD - Center for Regenerative Therapies Dresden, Technische Universität Dresden, Fetscherstraße 105, 01307 Dresden, Germany; 2 Genomics of Regeneration, CRTD - Center for Regenerative Therapies Dresden, Technische Universität Dresden, Fetscherstraße 105, 01307 Dresden, Germany; 3 German Center for Neurodegenerative Diseases (DZNE) Dresden, Arnoldstraße 18b, 01307 Dresden, Germany

**Keywords:** adult neurogenesis; hippocampus; stem cells; immune deficiency; regulatory T cells; cytokines; plasticity; learning and memory

## Abstract

CD4 ^+^ T cells provide a neuro-immunological link in the regulation of adult hippocampal neurogenesis, but the exact mechanisms underlying enhanced neural precursor cell proliferation and the relative contribution of different T helper (Th) cell subsets have remained unclear. Here, we explored the pro-proliferative potential of interleukin 17-producing T helper (Th17) cells, a developmentally and functionally distinct Th cell subset that is a key mediator of autoimmune neurodegeneration. We found that base-line proliferation of hippocampal precursor cells in a T cell-deficient mouse model of impaired hippocampal neurogenesis can be restored upon adoptive transfer with homogeneous Th17 populations enriched for myelin-reactive T cell receptors (TCR). In these experiments, enhanced proliferation was independent of direct interactions of infiltrating Th17 cells with precursor cells or neighboring cells in the hippocampal neurogenic niche. Complementary studies in immunocompetent mice identified several receptors for Th17 cell-derived cytokines with mRNA expression in hippocampal precursor cells and dentate gyrus tissue, suggesting that Th17 cell activity in peripheral lymphoid tissues might promote hippocampal neurogenesis through secreted cytokines.

## Background

In the hippocampus of the adult brain, neurogenesis originates from neural precursor cells residing in the subgranular zone of the dentate gyrus that proliferate and differentiate in response to intrinsic and extrinsic stimuli, allowing adaptation of the neuronal network to changing needs throughout life ^1, 2^. Besides innate immune mechanisms ^3, 4^, CD4 ^+^ T cells of the adaptive immune system promote adult hippocampal neurogenesis and convey functional benefits in reversal learning that have been related to adult neurogenesis ^5– 7^. However the molecular and cellular mechanisms underlying CD4 ^+^ T cell-mediated enhancement of adult neurogenesis have largely remained unclear. For example, whether or not the infiltration of myelin-reactive T cells into the CNS is a prerequisite for the proneurogenic activity of CD4 ^+^ T cells has been controversially discussed ^5, 6, 8, 9^. Additionally, the relative contribution of the activation or differentiation status of the CD4 ^+^ T cells to their proneurogenic activity remains to be determined. This includes CD4 ^+^ T cell-derived soluble factors that could either act directly on hippocampal precursor cells or promote precursor cell activity through indirect mechanisms, e.g. by acting on neighbouring cells within the neurogenic niche of the adult hippocampus.

Upon appropriate T cell and cytokine receptor signals, initially naïve CD4 ^+^ T cells can differentiate into different T helper (Th) cell subsets with distinct cytokine profiles and effector functions ^10– 12^. This includes interleukin-17 (IL-17)-producing Th17 cells that additionally express the orphan nuclear receptor ROR-γt. Besides mediating anti-microbial immunity at epithelial barriers ^13– 17^, ROR-γt ^+^ Th17 cells have been broadly linked to the pathogenesis of various autoimmune and chronic inflammatory conditions ^18– 23^, most notably demyelinating inflammatory disorders of the CNS, such as multiple sclerosis in humans and experimental autoimmune encephalomyelitis (EAE) in rodents. In EAE, a local reactivation of myelin-reactive Th17 cells that have crossed the blood-brain barrier initiates a cascade of neuroinflammatory responses, ultimately leading to demyelination in the CNS and neurodegeneration. More recent evidence suggests that there are different subsets of Th17 cells comprising a wide spectrum of effector phenotypes. Among these are nonpathogenic Th17 cells with regulatory properties that restrict tissue destruction during inflammatory responses and promote tissue remodeling and repair ^14, 24– 29^. This together with the broad expression of surface receptors for Th17-derived cytokines on both immune and non-immune cells ^15, 16, 30^, prompted us to assess the capacity of myelin-reactive Th17 cells to enhance precursor cell proliferation in an αβ T cell-deficient mouse model of impaired hippocampal neurogenesis. We concentrated on investigating proliferation as the most validated sub-process of adult hippocampal neurogenesis affected by T-cell alterations.

## Methods

### Mice

C57BL/6 mice were purchased from Janvier. Nestin ^GFP^ ^31^, TCRα ^−/−^^32^, and 2D2 mice ^33^ expressing a transgenic TCR recognizing amino acids 35-55 of myelin oligodendrocyte glycoprotein (MOG _35-55_), were on the C57BL/6 background. 2D2 mice additionally expressed a transgenic Foxp3 ^GFP^ reporter (2D2 × Foxp3 ^GFP^ ^34^). C57BL/6 and TCRα ^–/–^ mice were intercrossed to obtain heterozygous TCRα ^+/–^ F1 mice. All mice were housed at the Experimental Center of the Medizinisch-Theoretisches Zentrum (Technische Universität Dresden, Germany) under specific pathogen-free (SPF) conditions. They received food (standard mouse food „R/M-H“ from Ssniff Spezialitäten GmbH, Soest, Germany) and water ad libitum and lived on a light/dark cycle of 12 h/12 h with lights on at 8 am. Animal experiments were approved by the responsible regulatory authority at Regierungspräsidium Dresden (Approval numbers 24-9168.24-1/2008-5 and 24-9168.11-1/2008-12).

### Flow cytometry and cell sorting

Single cell suspensions of pooled spleen and lymph nodes (mesenteric and subcutaneous) from four- to six-week-old 2D2 × Foxp3 ^GFP^ mice were prepared using 70 µm cell strainers (BD). Monoclonal antibodies (mAbs) to CD4 (Monoclonal Rat IgG, GK1.5, BD Biosciences, Cat. No. 5532728), CD25 (Monoclonal Rat IgG, PC61, BD Biosciences, Cat. No. 551071) and CD62L (Monoclonal Rat IgG, MEL-14, eBioscience, Cat. No. 17-0621) and Pacific Blue-conjugated streptavidin were purchased from eBioscience or BD Biosciences. Before FACS, for some experiments, CD4 ^+^ cells were enriched using biotinylated mAbs against CD4, streptavidin-conjugated microbeads and an AutoMACS (Miltenyi Biotec). Intracellular ROR-γt expression was analyzed using the Foxp3 staining buffer set (eBioscience) and an anti-ROR-γt mAb (Monoclonal Rat IgG, AFKJS-9, eBioscience, Cat. No. 12-6988). Intracellular cytokine staining was performed using the Cytofix/Cytoperm kit and mAbs to IL-17 (Monoclonal Rat IgG, TC11-18H10.1, eBioscience, Cat. No. 51-7172-80) and IFN-γ (Monoclonal Rat IgG, XMG1.2, BD Biosciences, Cat. No. 554412). Samples were analyzed on a FACSCalibur or sorted using a FACSAria II or III (BD Biosciences). Data were analyzed using FlowJo software (Tree Star, Inc.).

### T cell culture and adoptive T cell transfer

T cells were cultured in IMDM medium, supplemented with 10% FCS (v/v), 1 mM sodium pyruvate, 1 mM HEPES, 2 mM Glutamax, 100 U/ml Penicillin-Streptomycin, 0.1 mg/ml Gentamicin, 0.1 mM non-essential amino acids, and 0.55 mM β-mercaptoethanol (all Invitrogen), at 37°C and 5% CO _2_. For Th17 differentiation *in vitro*, FACS-purified naïve CD4 ^+^ T cells (CD4 ^+^CD62L ^high^CD25 ^−^Foxp3 ^GFP−^) were cultured for one week in 24-well plates (0,5 × 10 ^6^ cells/ml) together with 20 Gy irradiated T cell-depleted C57BL/6 splenocytes at a 1:5 ratio, in the presence of soluble anti-CD3ε (2 μg/ml, Monoclonal Armenian Hamster IgG, 145-2C11, BD Biosciences, Cat. No. 550275), recombinant human TGF-β1 (1 ng/ml), murine IL-6 (50 ng/ml) (PeproTech), and neutralizing mAbs to IL-4 (10 μg/ml, Monoclonal Rat IgG, 11B11, eBioscience, Cat. No. 14-7041) and IFN-γ (10 μg/ml, Monoclonal Rat IgG, XMG1.2, eBioscience, Cat. No. 16-7311). After 2–3 days, cultures were supplemented with fresh cytokines. Murine IL-23 (10 ng/ml; R&D Systems) was added on day 4. Prior to flow cytometry of cytokine expression, Th17 differentiation cultures were briefly (4 h) restimulated on day 7 with 50 ng/ml Phorbol 12-myristate 13-acetate (PMA; Sigma-Aldrich) and 200 ng/ml Ionomycin (Iono; Calbiochem), in the presence of 10 μg/ml brefeldin A (BFA; Sigma-Aldrich). On day 7 of Th17 differentiation cultures, 4 × 10 ^6^ cells/200 μl PBS were injected i.v. into six-week-old TCRα ^−/−^ recipients. Control mice received PBS only. Adoptively transferred CD4 ^+^ T cells were tracked by flow cytometry after 2 weeks in the peripheral blood of recipients, as indicated.

### BrdU administration and immunohistochemistry

Eight-week-old mice received 3 consecutive i.p. injections of BrdU (50 mg/kg body weight in 100 μl NaCl; Sigma-Aldrich) at intervals of 6 hours. Twenty-four hours after the first injection, mice were killed with an overdose of anesthetics and perfused transcardially, first with ice-cold saline and then with 4% paraformaldehyde (Sigma-Aldrich). The brains were removed from the skull, postfixed overnight, washed with PBS and cryoprotected for ≥ 3 days in a 30% sucrose solution. Free-floating, 40 μm coronal sections were obtained on a freezing microtome (Leica SM2010R) and stored at 4°C. Immunohistochemistry was performed on 1-in-6 series of free-floating sections of each brain as previously described ^35^. To visualize the immune reaction we used the peroxidase method (ABC-Elite; Vector Laboratories) with biotinylated anti-rat and anti-rabbit antibodies (Jackson ImmunoResearch) and nickel-intensified diaminobenzidine (DAB; Sigma-Aldrich) as chromogen. Primary antibodies were rat anti-BrdU (Monoclonal Rat IgG, BU1/75 (ICR1), AbD Serotec, Cat. No. MCA2060) or polyclonal rabbit anti-CD3 (Abcam, Anti-CD3 antibody, ab 5690). Sections were mounted on gelatine-coated slides, air-dried, incubated in Neoclear (Merck) for 90 min and coverslipped. BrdU ^+^ cells in the granule cell layers and within two cell diameters below in the subgranular zone of the dentate gyrus on both sides were counted exhaustively throughout the rostro-caudal extension of the dentate gyrus by an observer blind to the treatment conditions on a light microscope (Leica DM750, 40x objective). Numbers of BrdU ^+^ cells in the selected coronal sections of each brain were multiplied by 6 as an estimate of total BrdU ^+^ cell numbers in both dentate gyri.

### Dentate gyrus microdissection and neural precursor cell culture

For RNA isolation, dentate gyri of seven- to eight-week-old Nestin ^GFP^ mice were dissected as described before ^36^. For hippocampal precursor cell cultures from microdissected dentate gyri of adult seven- to eight-week-old C57BL/6 mice, tissue dissection, digestion and cell enrichment were performed as previously described ^37, 38^. After enrichment, 1 × 10 ^4^ cells/cm ^2^ were cultured in poly-D-lysine- and laminin-coated (Sigma-Aldrich and Roche, respectively) T25 cell culture flasks (TPP) in proliferation medium, consisting of Neurobasal Medium supplemented with B27, Glutamax and 50 U/ml Penicillin-Streptomycin (all Invitrogen), as well as 20 ng/ml human Fibroblast Growth Factor-basic (FGF-2) and 20 ng/ml human Epidermal Growth Factor (EGF; both PeproTech). Every other day, 75% of the medium was replaced by fresh medium. Cells were passaged, when 80% of confluence was reached.

### RNA isolation and RT-PCR

For mRNA expression analysis of cells from microdissected dentate gyri, the tissue was passed several times through a 25-gauge needle in RLT buffer (QIAGEN) supplemented with 1% β-Mercaptoethanol (Bio-Rad). For mRNA expression analysis of isolated neural precursor cells, cultured cells were detached from the flask surface with Accutase (PAA) and washed with PBS prior to lysis in RLT buffer. Total RNA was extracted using the RNeasy Mini kit according to the manufacturer’s protocol (Qiagen), including on-column DNase I digestion to minimize genomic DNA contaminations. For real-time RT-PCR, cDNA was synthesized using SuperScript II reverse transcriptase (Invitrogen). cDNA was analyzed in duplicates using a Mastercycler ep realplex thermal cycler (Eppendorf), the QuantiFast SYBR Green PCR kit (Qiagen), and primers listed in Table 1. With the exception of GAPDH ^39^, primers were designed using NCBIPrimer-BLAST ( http://www.ncbi.nlm.nih.gov/tools/primer-blast/). Relative mRNA expression was calculated using the ΔCt method and GAPDH as housekeeping gene. Only mRNAs with an expression below ΔCt = 15 were considered to be expressed. PCR specificity was confirmed employing melting curve analysis and gel-electrophoresis of PCR products.

### Statistical analysis

**Figure 1.**
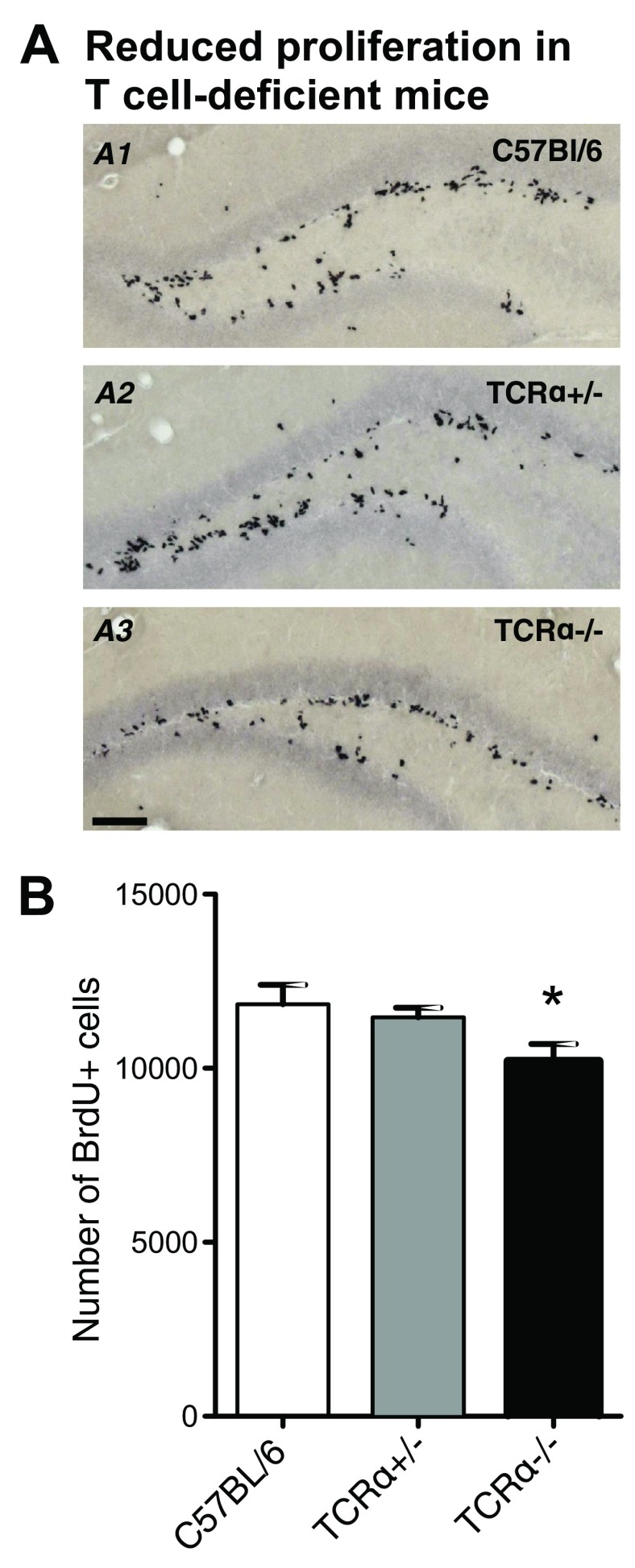
Impaired base-line proliferation in the hippocampus in the absence of αβ T cells. ( **A**) Representative BrdU immunohistochemistry of the hippocampal dentate gyrus from eight week-old wild-type (A1), TCRα ^+/−^ (A2) and TCRα ^−/−^ (A3) mice, 24 hours after the first of 3 consecutive BrdU injections. Scale bar, 100 μm. ( **B**) Quantification of BrdU ^+^ cells in the dentate gyrus of wild-type (n = 6), TCRα ^+/−^ (n = 7) and TCRα ^−/−^ (n = 8) mice. All numbers are mean ± SEM. ANOVA, * p < 0.05.

**Figure 2.**
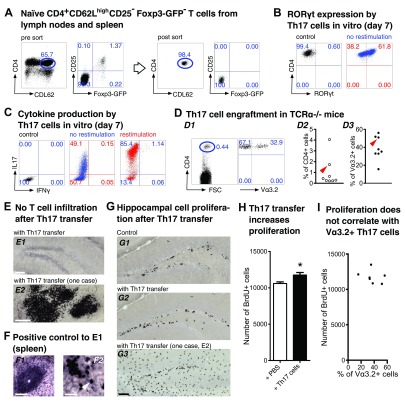
Th17-polarized CD4 ^+^ T cells are sufficient to promote hippocampal precursor cell proliferation in adult TCRα ^–/–^ mice. ( **A**– **C**) Th17 polarization *in vitro*. ( **A**) Flow cytometry of encephalitogenic CD4 ^+^ T cells. Dot plots show pre-sort (left) and post-sort (right) analysis of naïve T cells (CD4 ^+^CD62L ^high^CD25 ^−^Foxp3 ^GFP−^) from pooled spleen and lymph nodes of 2D2 x Foxp3 ^GFP^ mice. FACS-purified T cell populations were cultured under Th17-polarizing conditions, as described in the Methods section. On day 7, efficiency of Th17 cell differentiation was confirmed by intracellular flow cytometry of ( **B**) the Th17 transcription factor ROR-γt and ( **C**) the Th17 and Th1 signature cytokines IL-17 and IFN-γ, respectively. ( **D**– **I**) Impact of adoptive Th17 cell transfer on hippocampal precursor cell proliferation in TCRα ^−/−^ mice. ( **D**) On day 7, 4 × 10 ^6^ total cells from Th17 polarization cultures were injected i.v. into adult TCRα ^−/−^ mice. (D1) Dot plots show representative flow cytometry of CD4 ^+^ T cells in peripheral blood of recipient mice that express the Vα3.2 subunit of the transgenic 2D2 TCR, two weeks after adoptive transfer. (D2) and (D3) Graphs show composite percentages of total CD4 ^+^ T cells (D2) and MOG _35-55_-reactive Vα3.2 ^+^ T cells among gated CD4 ^+^ T cell populations (D3) from peripheral blood of recipient mice. The arrowheads in (D2) and (D3) highlight an individual mouse that exhibited immune cell infiltrations in the brain (see below). Numbers in dot plots in ( **A**– **D**) indicate the percentage of cells in the respective quadrant or gate. ( **E**, **F**) Anti-CD3 immunohistochemistry. ( **E**) Immunohistochemistry of the dentate gyrus of TCRα ^−/−^ recipient mice for the pan-T cell marker CD3, two weeks after adoptive Th17 cell transfer. Infiltrating CD3 ^+^ T cells were found to be below the level of detection in all mice analyzed (E1, scale bar, 100 μm), with the exception of an individual recipient mouse that exhibited CD3 ^+^ T cell and other immune cell infiltrations in some brain areas, including the hippocampus (E2, scale bar, 100 μm). ( **F**) Anti-CD3 immunohistochemistry of the spleen from wild-type C57BL/6 mice was included as a positive control (F1, scale bar, 100 μm; F2, scale bar, 25 μm). The arrowhead in (F2) indicates an individual CD3 ^+^ T cell. ( **G**, **H**) Quantification of hippocampal cell proliferation. ( **G**) BrdU immunohistochemistry of the dentate gyrus of TCRα ^−/−^ mice, which had been injected with either (G1) PBS or (G2) Th17 cells two weeks earlier, was performed 24 hours after the first of 3 consecutive BrdU injections. (G3) depicts the dentate gyrus of the mouse exhibiting immune cell infiltrations (see E2). Scale bar, 100 μm. ( **H**) Quantification of BrdU ^+^ cells in the dentate gyrus of TCRα ^−/−^ mice injected with either PBS (n = 7) or Th17 cells (n = 7). All numbers are mean ± SEM. t-test, * p < 0.05. ( **I**) Scatter diagram to visualize a possible relationship between cell proliferation in the dentate gyrus and the percentage of MOG _35-55_-reactive Vα3.2 ^+^ T cells among CD4 ^+^ T cells in the peripheral blood of recipient mice two weeks after adoptive transfer. No statistically significant correlation was found.

Statistical analysis was performed with GraphPad Prism 5 software and the GraphPad web calculator ( http://www.graphpad.com/quickcalcs/). A two-tailed unpaired Student’s t-test was used for analysis of the experiments shown in Figure 2. Data from the experiments shown in Figure 1 were analyzed with ANOVA followed by Dunnett’s Multiple Comparison Test. Differences were considered statistically significant at p < 0.05.

## Results

**Figure 3.**
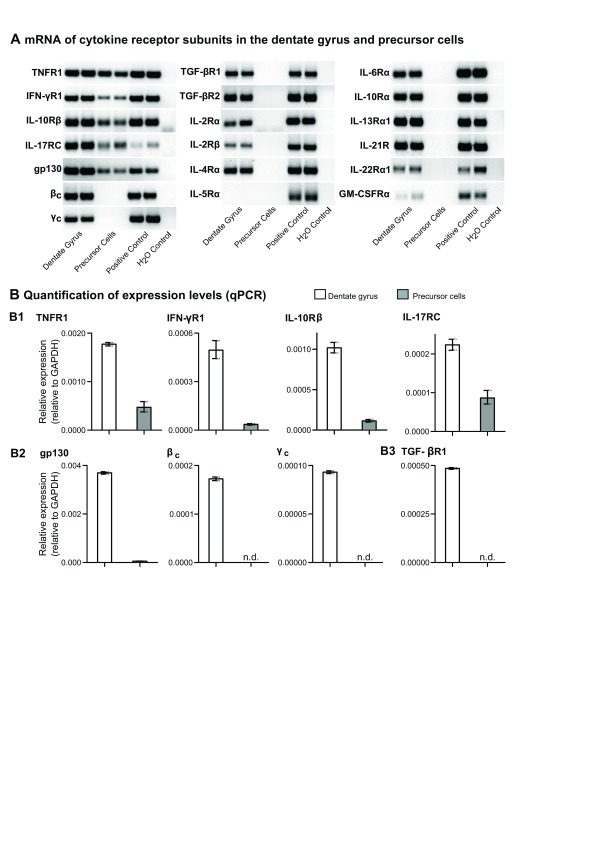
Cytokine receptor expression in the neurogenic niche of the hippocampal dentate gyrus. Freshly microdissected tissue and cultured neural precursor cells from the dentate gyrus of adult Nestin ^GFP^ and C57BL/6 wild-type mice were subjected to mRNA expression analysis of T cell-relevant cytokine receptor genes by real-time RT-PCR, as indicated. ( **A**) Gel electrophoresis of RT-PCR duplicate samples. For details on indicated cytokine receptor genes, see below. Total cells from spleen and pooled lymph nodes were included as positive control. ( **B**) Quantification by real-time RT-PCR. Relative mRNA expression values of indicated genes encoding cytokine receptor subunits, as revealed by quantitative RT-PCR using GAPDH for normalization. Only mRNAs with an expression below ΔCt = 15 were considered to be expressed (n.d., not detected). Shown are mean values ± range of duplicate samples. (B1) Receptor subunits for essential T cell effector cytokines, including Th17 cell-derived cytokines, were expressed in the dentate gyrus as well as isolated precursor cells. TNFR1, IFN-γR1, IL-10Rβ and IL-17RC are components of the receptors for TNF-α, IFN-γ (Th1), IL-10 (Treg/Th2) as well as IL-22, IL-17A and IL-17F (Th17). (B2) Shared receptor subunits of class I cytokine receptors (glycoprotein 130, gp130; common beta subunit, β _c_; common gamma subunit, γ _c_) were expressed in the dentate gyrus but, with the exception of gp130, not by the isolated precursor cells. Similar results were obtained for TGF-βR1, TGF-βR2, IL-2Rα (ΔCt = 17,86), IL-2Rβ (ΔCt = 16.75), IL-4Rα, IL-6Rα, IL-10Rα, IL-13Rα1, IL-21R, IL-22Rα1 (ΔCt = 18.78) and GM-CSFRα (ΔCt = 19.22). As a representative example, relative mRNA expression values for TGF-βR1 are shown (B3). Expression of the cytokine receptor mRNA for IL-5Rα could be detected neither in microdissected tissue nor in cultured precursor cells from the dentate gyrus.

Hippocampal neurogenesis data from T helper 17 cell-deficient and control mice *Dataset 1a.* Baseline proliferation levels of hippocampal precursor cells in TCRα ^–/–^ and TCRα ^+/–^ mice compared to C57BL/6 controls. The values shown are the numbers of BrdU ^+^ cells in the bilateral dentate gyrus of each single animal in the three different groups. The data refer to the results presented in Figure 1.
*Dataset 1b.* Proliferation levels of hippocampal precursor cells in TCRα ^–/–^ mice after adoptive Th17 cell transfer compared to sham-injected TCRα ^–/–^ controls. The values shown are the numbers of BrdU ^+^ cells in the bilateral dentate gyrus of each single animal in the two different groups. The numbers in brackets represent the percentages of total CD4 ^+^ T cells/Vα3.2 ^+^ T cells among gated CD4 ^+^ T cell populations from the peripheral blood of recipient mice two weeks after adoptive Th17 cell transfer. The data refer to the results presented in Figure 2. The last entry in the left-hand column was excluded from statistical analysis due to determination as significant outlier by Grubbs’ test.
*Dataset 1c.* Quantification of expression levels of cytokine receptor genes in cells of the neurogenic niche of the hippocampus and hippocampal precursor cells from cell culture by real-time RT-PCR. Shown are mean values for ΔCt and the relative expression levels ± range of duplicate samples, using the ΔCt-method and GAPDH for normalization. The data refer to the results presented in Figure 3. Click here for additional data file. Copyright: © 2017 Niebling J et al. 2017 Data associated with the article are available under the terms of the Creative Commons Zero "No rights reserved" data waiver (CC0 1.0 Public domain dedication).

### Impaired base-line proliferation of hippocampal precursor cells in adult TCRα ^–/–^ mice

We first assessed steady-state levels of cell proliferation in the hippocampal dentate gyrus of adult, 8-week-old TCRα ^–/–^ mice, which are characterized by a complete lack of αβ T cells (both CD4 ^+^ and CD8 ^+^) due to targeted deletion of the gene encoding the TCRα subunit (TCRα ^–/–^). For *in vivo* labeling of dividing cells, TCRα ^–/–^ mice received three consecutive i.p. injections of the thymidine analog bromodeoxyuridine (BrdU) at intervals of six hours. In these experiments, age-matched cohorts of fully immunocompetent TCRα ^+/–^ and C57BL/6 wild-type mice were included for comparison. Twenty-four hours after the first BrdU injection, experimental mice were subjected to immunohistochemical quantification of BrdU ^+^ cells in the dentate gyrus ( Figure 1A). Consistent with a published study in TCRα ^–/–^ mice based on endogenous Ki67 expression as proliferation marker ^7^, we found that TCRα ^–/–^ mice (10205 ± 492 BrdU ^+^ cells, n = 8) exhibited significantly reduced levels of proliferation compared to C57BL/6 wild-type mice (11843 ± 556, n = 6; ANOVA, F (2, 18) = 3.698, p < 0.05; Figure 1B). TCRα ^+/–^ mice (11469 ± 273, n = 7) ranged between controls and knockouts. Overall, these results are in agreement with our previous observation that CD4 ^+^ T cells provide a neuro-immunological link in the base-line regulation of hippocampal precursor cell activity ^6^.

### Th17 cell-mediated restoration of proliferation in adult TCRα ^–/–^ mice

To assess the impact of myelin-reactive Th17 cells on proliferation *in vivo*, we employed adoptive T cell transfers using six-week-old mice and quantified BrdU ^+^ cells in the hippocampus of TCRα ^-/-^ recipients two weeks later. For the generation of Th17 cells, naïve CD4 ^+^ T cells (CD4 ^+^CD62L ^high^CD25 ^−^Foxp3 ^GFP−^) carrying the MOG _35-55_-specific 2D2 TCR as a transgene were FACS-purified from peripheral lymphoid tissues of four- to six-week-old 2D2 × Foxp3 ^GFP^ mice ( Figure 2A) and cultured under T cell stimulatory conditions that promote efficient differentiation into Th17 cells with a ROR-γt ^+^IL-17 ^+^ phenotype ( Figure 2B and C). As expected based on previous observations with differentiated Th17 cells *in vitro*, these cultures exhibited limited IFN-γ production ( Figure 2C).

On day 7 of Th17 differentiation cultures, 4 × 10 ^6^ total cells were injected i.v. into adult, age-matched cohorts of TCRα ^–/–^ mice. TCRα ^–/–^ mice that received PBS only were included as controls. Two weeks later, small populations of adoptively transferred CD4 ^+^ T cells could be detected in the peripheral blood of recipient mice ( Figure 2D). In these experiments, significant proportions of CD4 ^+^ Th17 cells expressed the MOG _35-55_-specific 2D2 TCR transgene (ranging from 15.6 % to 56.1%), as judged by flow cytometry of the TCRα subunit of the transgenic 2D2 TCR, employing anti-Vα3.2 mAbs ( Figure 2D). Importantly, throughout the observation period, TCRα ^–/–^ recipients of *in vitro* generated Th17 cells appeared phenotypically healthy and exhibited no clinical symptoms of EAE. Consistently, immunohistochemistry for the pan-T cell marker CD3 revealed that infiltrating Th17 cells in the dentate gyrus were below the detection level in all mice analyzed ( Figure 2E and F), with the exception of an individual recipient with immune cell infiltrates, including some CD3 ^+^ T cells ( Figure 2E2).

Two weeks after adoptive T cell transfer, cell proliferation in the dentate gyrus of TCRα ^–/–^ recipients was assessed by immunohistochemical quantification of BrdU ^+^ cells, as described above. After applying Grubbs’ outlier test, one animal in the control group with exceptionally high numbers of BrdU-positive cells was excluded from further analysis. The results showed that TCRα ^–/–^ recipients of Th17 cells exhibit significantly increased numbers of BrdU ^+^ cells in the hippocampal dentate gyrus (11758 ± 347, n =7), as compared to control-injected TCRα ^–/–^ mice (10602 ± 214, n = 7; t-test, p < 0.05; Figure 2G and H). Thus, populations of Th17 cells enriched for myelin-reactive TCRs appear sufficient to restore impaired base-line proliferation of hippocampal precursor cells in adult TCRα ^–/–^ recipient mice, in the absence of T cell infiltration and direct interaction with neural precursor cells or cellular components of the hippocampal neurogenic niche such as microglia. Consistently, the enhanced proliferative activity of precursor cells did not correlate with the proportion of Vα3.2 ^+^ Th17 cells that accumulated in recipient mice (Pearson’s r = –0.28, p = 0.538, 95% CI –0.85 to 0.60, n = 7; Figure 2I).

### Cytokine receptor expression in the neurogenic niche of immunocompetent mice

Besides the signature cytokines IL-17A and IL-17F, ROR-γt ^+^ Th17 cells have been reported to produce a variety of cytokines such as TNF-α, IFN-γ, IL-9, IL-10, IL-21 and IL-22. In a first attempt to provide insight into possible mechanisms underlying the enhancement of hippocampal precursor cell proliferation by Th17 cell-secreted cytokines, we assessed expression levels of mRNAs encoding relevant cytokine receptors in the neurogenic niche. To this end, we performed quantitative RT-PCR analysis of freshly microdissected dentate gyrus as well as isolated precursor cells from the dentate gyrus of adult, immunocompetent C57BL/6 mice ( Figure 3).

This approach identified several subunits of receptors for Th17-derived cytokines with detectable mRNA expression levels in both total dentate gyrus tissue and isolated precursor cells ( Figure 3A and B), namely IL-17 receptor C (IL-17RC), tumor necrosis factor R1 (TNFR1), interferon-gamma R1 (IFN-γR1) as well as IL-10R beta (IL-10Rβ), a common subunit involved in the formation of the receptors for IL-10 and IL-22 ^40^. Next, we extended our analysis to the type I cytokine receptor family (glycoprotein 130: gp130, CD130; common γ subunit: γc, CD132; common β subunit: βc, CD131), which is involved in the formation of more than 20 different cytokine receptors. In these experiments, mRNA expression of all three receptor family members (gp130, βc, γc) could be detected in total dentate gyrus tissue ( Figure 3A and B). Furthermore, mRNA encoding gp130, a subunit shared between the receptors for cytokines such as IL-6, leukemia inhibitory factor (LIF) and ciliary neurotrophic factor (CNTF), was also expressed in isolated neural precursor cells. Interestingly, IL-6, LIF and CNTF directly affect the differentiation of adult hippocampal precursor cells *in vitro* ^41– 44^.

Cytokine receptor subunits with detectable mRNA expression levels in microdissected total tissue but not in isolated precursor cells from the dentate gyrus included transforming growth factor beta receptor 1 and 2 (TGF-βR1 and TGF-βR2), IL-2R alpha and beta (IL-2Rα and IL-2Rβ), IL-4Rα, IL-10Rα, IL-13Rα1, IL-21R, IL-22Rα1 and granulocyte-macrophage colony stimulating factor receptor alpha (GM-CSFRα). Among these the ΔCt-values for IL-2Rα (ΔCt = 17,86), IL-2Rβ (ΔCt = 16.75), IL-22Rα1 (ΔCt = 18.78) and GM-CSFRα (ΔCt = 19.22) were found to be below the chosen cut-off (ΔCt < 15). Nevertheless, in all of these cases a specific product could be detected by gel electrophoresis of RT-PCR samples ( Figure 3A). Additionally, and in contrast to previous reports on IL-6Rα mRNA expression in dentate gyrus-derived precursor cells ^42^, we found IL-6Rα mRNA to be expressed in whole dentate gyrus but not in isolated neural precursor cells. The underlying reason for this apparent discrepancy between studies remains to be determined, but may include methodological differences in the preparation and/or purity of isolated precursor cells. Lastly, among the cytokine receptor subunits whose expression was analyzed in the present study, we failed to detect mRNA expression for IL-5R alpha (IL-5Rα) in both microdissected tissue and isolated precursor cells ( Figure 3A).

## Discussion

Previous studies on mice with transgenic expression of a myelin-specific TCR on CD4 ^+^ T cells ^5^ and a non-TCR transgenic mouse model of MOG-inducible EAE ^9^ have provided the first evidence that encephalitogenic CD4 ^+^ T cell activity can promote hippocampal precursor cell proliferation and adult neurogenesis. Here, we have extended these observations and show that, in the absence of autoimmune neuroinflammation, small numbers of myelin-reactive CD4 ^+^ T cells with a ROR-γt ^+^IL-17 ^+^ phenotype are sufficient to restore base-line proliferation of hippocampal precursor cells in TCRα ^–/–^ mice that lack endogenous αβ T cells.

Mechanistically, and consistent with the proneurogenic activity of non-infiltrating CD4 ^+^ T cells with a polyclonal TCR repertoire ^6, 7^, the overall absence of immune cell infiltrations in our Th17 adoptive transfer model emphasizes that direct cell-cell interaction is not a prerequisite of enhanced Th17-mediated hippocampal precursor cell proliferation. Alternatively, Th17 cells residing in peripheral lymphoid tissues outside the brain may secrete cytokines that are actively transported across the blood-brain barrier ^45, 46^ and act on the hippocampal neurogenic niche to promote precursor cell proliferation. Indeed, it is becoming increasingly clear that the impact of inflammatory cytokines on hippocampal neurogenesis appears much more context-dependent than anticipated based on previous studies highlighting overall detrimental effects ^3, 4, 47– 49^. Factors that influence the impact of inflammatory cytokines on neurogenesis include the administration route and local cytokine concentrations, the strength and duration of enhanced cytokine receptor signalling as well as the target cell within the neurogenic niche. While the present study suggests that several receptors for Th17 cell-derived cytokines (TNF-α, IFN-γ, IL-17, IL-22) are expressed on hippocampal precursor cells as well as neighbouring cells in the dentate gyrus, it will be important to investigate whether the pattern of expressed cytokine receptors observed in mice under physiological baseline conditions is subject to differential regulation in response to intrinsic or extrinsic stimuli. Another important, unresolved question is whether the proneurogenic effect of Th17 cells can be attributed to an individual inflammatory cytokine or is rather mediated by the combined action of different Th17-cell derived factors.

At present, cytokines with reported proneurogenic potential in hippocampal neurogenesis include IFN-γ ^50, 51^, TNF-α ^52, 53^, TGF-β ^54^, CNTF ^44^ as well as IL-1β and IL-6 ^42, 55^. Interestingly, the Th17 signature cytokine IL-17 has recently been found to increase neurite outgrowth from adult postganglionic sympathetic neurons, a process that required NFkB activation ^28^. Importantly, the NFkB pathway, which is shared between many cytokine receptor signaling pathways, has previously been implicated in the regulation of neural precursor cell proliferation and differentiation ^56, 57^. Clearly, future studies are warranted to directly address a putative role of IL-17 and the NFkB pathway in hippocampal proliferation and neurogenesis.

In summary, the present study exemplifies that the TCRα ^–/–^ mouse represents a suitable experimental model to assess the proneurogenic potential of homogeneous Th cell populations that had been generated under well-defined *in vitro* conditions. This is likely to facilitate mechanistic studies on the relative contribution of various CD4 ^+^ Th cell subsets (Th1, Th2, Th17 etc.) to the regulation of adult hippocampal neurogenesis.

## Data availability

The data referenced by this article are under copyright with the following copyright statement: Copyright: © 2017 Niebling J et al.

Data associated with the article are available under the terms of the Creative Commons Zero "No rights reserved" data waiver (CC0 1.0 Public domain dedication). 

F1000Research: Dataset 1. **Hippocampal neurogenesis data in T helper 17 cell-deficient and control mice**, http://dx.doi.org/10.5256/f1000research.4439.d157588 ^58^
