# Supplementary material for: Myelin-specific T helper 17 cells promote adult hippocampal neurogenesis through indirect mechanisms
Source: F1000Res. 2017 Apr 7;3:169. Originally published 2014 Jul 24. [Version 2] doi: 10.12688/f1000research.4439.2 (PMC4215755; doi:10.12688/f1000research.4439.2)
Supplement: Peer review history

# Peer review: Referee response for version 2

**DOI:** 10.5256/f1000research.12255.r21904

**Published:** 2017-04-18

**Authors:** Francis G. Szele

**Affiliations:** 1 Department of Physiology, Anatomy and Genetics, University of Oxford, Oxford, UK

I have read the revised paper and approve it.

I have read this submission. I believe that I have an appropriate level of expertise to confirm that it is of an acceptable scientific standard.

# Peer review: Referee response for version 2

**DOI:** 10.5256/f1000research.12255.r21677

**Published:** 2017-04-10

**Authors:** Stefano Pluchino

**Affiliations:** 1 Department of Clinical Neurosciences, John Van Geest Centre for Brain Repair, University of Cambridge, Cambridge, UK

The authors have now addressed in full my previous criticisms and this revised version of the paper is of an acceptable standard.

I have read this submission. I believe that I have an appropriate level of expertise to confirm that it is of an acceptable scientific standard.

# Peer review: Referee response for version 1

**DOI:** 10.5256/f1000research.4750.r5570

**Published:** 2014-08-26

**Authors:** Francis G. Szele

**Affiliations:** 1 Department of Physiology, Anatomy and Genetics, University of Oxford, Oxford, UK

The link between the immune system and neurogenesis has become increasingly interesting with some papers showing positive and others showing negative effects in health and disease. It is likely that different subsets of immune cells, and different states of activation are in part responsible for these discrepancies. This short paper begins to address the need for detailed examination of these immune cell subsets by showing that systemic adoptive transfer of myelin reactive T17 cells into a mouse model with T cell deficiency rescues proliferation in the hippocampal dentate gyrus. This appears to occur without the need for direct cell contact since the majority of mice did not have significant numbers of transplanted T cells in the hippocampus.

1. The decrease in TCRa ^-/-^ DG proliferation, though statistically significant, is rather small and one wonders whether these differences would translate into functional differences. Is there also increased neurogenesis? The paper would benefit from additional experiments showing neurogenic changes are paralleled by behavioral changes.
2. Could it be that T17 cells migrated into and then left the DG within the first week after adoptive transfer? What are the kinetics of T cell immigration and emigration into the DG?
3. Did you exclude the animal (Fig. 2E2) with significant T cell infiltration from your analysis?
4. The authors present a hypothesis that T cell derived cytokines affect DG neurogenesis. It would be interesting to examine cytokine receptor levels in TCRa ^-/-^ mice.

Minor Points:

- Please spell out TCR the first time it is used.
- Scull should be "skull”.
- Fig. 3 title *“mRNA of cytokine receptor chains in the dentate gyrus and precursor cells*”; I recommend substituting “chain” with “subunit”.

I have read this submission. I believe that I have an appropriate level of expertise to confirm that it is of an acceptable scientific standard, however I have significant reservations, as outlined above.

# Response

**Published:** 2017-04-03

**Authors:** Gerd Kempermann

**Affiliations:** German Center for Neurodegenerative Diseases (DZNE) Dresden, Germany

1.  The decrease in TCRa-/- DG proliferation, though statistically significant, is rather small and one wonders whether these differences would translate into functional differences. Is there also increased neurogenesis? The paper would benefit from additional experiments showing neurogenic changes are paralleled by behavioral changes.

- Impaired precursor cell proliferation has been observed in different animal models of adaptive immune deficiency. While this effect appeared to be rather discrete using TCRα ^-/-^mice in the present study and the work by Huang and colleagues ^7^, more pronounced results could be obtained in severe combined immune deficiency (SCID) and RAG1 ^-/-^mice, respectively ^5,6^. Since the relative decrease in proliferation in RAG1 ^-/-^and TCRα ^-/-^mice was fairly comparable using Ki67 as a marker of proliferation ^7^, it is possible that the different methodologies in addition to the genetic background of the animals caused the discrepancies between the mentioned studies.

The question on whether our observed increased proliferation after adoptive transfer results in more new-born neurons together with the rational to restrict our analysis to proliferation as one component of adult neurogenesis has been discussed above (our response to the 1. main comment of the first Reviewer, S. Pluchino). In brief, our aim was to follow up on previous studies showing increased proliferation and, likely as a consequence, more neurons after CD4 ^+^ T-cell reconstitution ^5,6,7^ and further define the relevant T-cell subtypes.

 The functional relevance of T cells as a positive regulation factor of hippocampal neurogenesis is still a matter of debate. Due to resource limitations, we were not able to extend our observations on hippocampus-dependent learning processes, which can be objectified in the Morris water maze (MWM). In this regard, we can only refer to previous work done by our group, in which CD4-depleted mice had shown an impaired performance at least in the reversal learning phase of the MWM ^6^.

 2.  Could it be that T17 cells migrated into and then left the DG within the first week after adoptive transfer? What are the kinetics of T cell immigration and emigration into the DG?

- Clearly we cannot exclude a transient infiltration of Th17 cells into the CNS during the two weeks between adoptive transfer and analysis. The absence of clinical symptoms during the observation period, however, argues against a neuroinflammatory process. As already stated in the manuscript, it is per se unlikely that a relevant amount of immune cells infiltrate into the brain parenchyma across an intact BBB under physiological conditions (Engelhardt & Ransohoff, 2005; Prendergast & Anderton, 2009). In murine EAE the first signs of clinical disease can be observed within a period of four up to >100 days post induction depending on the genetic background of the host animals and the mode of disease induction (Miller et al.. 2010). Demyelination and inflammation in the CNS thereby largely reflect the clinical expression of the disease (Pachner, 2011). Using intravital imaging in a rat model of passive EAE, an infiltration of transferred T cells into the brain parenchyma could be seen as early as on day three after transfer (Bartholomäus et al. 2009). By the adoptive transfer of polyclonal as well as non-activated myelin-specific T cell populations into immunodeficient host animals and subsequent CD3 immunohistochemistry, our group could, however, exclude an early immigration of T cells into the brain within the first four days following injection, at least under the chosen experimental conditions (Wolf et al., 2009b; unpublished data).

3.  Did you exclude the animal (Fig. 2E2) with significant T cell infiltration from your analysis?

- Although the cell infiltrations found in the CNS of this specific mouse were limited to only a few brain regions, they affected some parts of the hippocampus as well. As it can be seen in Figure 2G3, this would have certainly prevented a correct and unbiased quantification of cellular proliferation, which is why the animal concerned was excluded from our analysis.

4.  The authors present a hypothesis that T cell derived cytokines affect DG neurogenesis. It would be interesting to examine cytokine receptor levels in TCRa-/- mice.

- Even though several other cell types within the brain, such as astrocytes and microglia, were found to produce a plethora of different effector cytokines (Sawada et al., 1995; Gonzalez-Perez et al., 2012), there seems to be no doubt that an absence of T cells, as a major source of cytokines in the organism, could potentially lead to differential expression of cytokine receptors in the putative target cells of the neurogenic niche. Quantitative RT-PCR could be adopted to verify differences in the mRNA expression levels of cytokine receptor genes between TCRα ^-/-^and immunocompetent control mice. Still, although this kind of experimental approach might eventually contribute to a better understanding of the general picture of a T cell-mediated regulation of adult hippocampal neurogenesis, it would probably only allow very imprecise interpretation of the underlying molecular signalling, which is why we do not consider it to be a prerequisite for the conclusions drawn in the current manuscript.

Minor Points:

 •   Please spell out TCR the first time it is used.

 •   Scull should be "skull”.

 •   Fig. 3 title “ *mRNA of cytokine receptor chains in the dentate gyrus and precursor cells*”; I recommend substituting “chain” with “subunit”.

- These points have been addressed.

# Peer review: Referee response for version 1

**DOI:** 10.5256/f1000research.4750.r5572

**Published:** 2014-08-05

**Authors:** Carlos P. Fitzsimons

**Affiliations:** 1 Center for Neuroscience, Swammerdam Institute for Life Sciences,, University of Amsterdam, Amsterdam, Netherlands

In this paper, Niebling *et al.*, study the regulation of hippocampal neurogenesis by interleukin 17-producing T helper (Th17) cells. Using a T-cell deficient mouse model with impaired base-line levels of hippocampal neurogenesis, they found that the neurogenesis impairment present in these animals can be restored after transfer of homogenous populations of Th17 cells, in a way that was independent of direct interaction between the transferred Th17 cells and cells of the dentate gyrus. From these results, they conclude that Th17 cells may promote hippocampal neurogenesis via secreted cytokines.

The manuscript is well written and easy to read, the title is descriptive of the content and the abstract provides a clear summary of the article.

The experiments are clearly designed and information on how the data has been analyzed is provided.

The main conclusions made by the authors are supported by the data presented in the manuscript’s figures.

Main Comments:

1. Some details in the description of the methods are unclear. Specifically, the age of the animals used in the experiments is not clearly stated in the corresponding methods section. Although eight weeks old is mentioned in legend to figure 1 this should be clarified in the methods section because age is a strong determinant of base-line hippocampal neurogenesis. Were animals transcardially perfused until complete elimination of blood cells before fixation? The authors should clarify this point to facilitate the reader’s interpretation of the data presented in figure 3, discarding blood cell contamination in dentate gyrus microdissected tissue samples, which could be an alternative interpretation for the differences in cytokine receptor expression in tissue vs. cultured precursor cells (last paragraph of the results section).
2. The authors suggest that one of the limitations of their study is the lack of direct characterization of the specific factor/cytokine(s) responsible for the cell-cell interaction-independent promotion of hippocampal neurogenesis they observe. However, it could have been very interesting to see some experimental effort made to support the hypothesis that the effects of Th17 cells are mediated by specific cytokines produced by these cells. This could have been done in vitro, using their cultured precursor cells, providing a stronger first proof-of-principle for the important concept they propose.
3. In the adoptive T cell transfer experiments, control mice were injected with PBS only. Although this experimental approach provides a suitable negative control, one wonders whether the injection of other non-IL17 producing T cells (e.g. naive CD4+ differentiated into Th1 or Th2 cells) could have been a more sensible negative control for these experiments, given the specific immune functions of these different T cell sets, which are highlighted by the authors in the last sentence of their discussion. Perhaps the authors could comment on the limitations of their experimental approach in this respect?
4. The discussion could benefit from a short paragraph including a more detailed discussion of possible pathological implications of the observations described in the paper. For example, other authors have recently found that hippocampal neurogenesis is enhanced in an arthritis model in rat ( Leuchtweis *et al.*, 2014). Perhaps another interesting point of discussion could be the possible implications for depression, a disease commonly linked to alterations in hippocampal neurogenesis and possibly linked to immune function ( Eyre and Baune, 2012).
5. The data presented in dataset 1 seems compatible with the figures presented with the article. However, the data could be presented in a format more easily understandable for the reader. i.e. Dataset 1a is labelled “C57BL/6,TCR?+/?,TCR??/?”, while is should presumably be “C57BL/6, TCRα ^+/-^, TCRα ^−/−^” and the data in rows 8 and 9 seems to be in an odd format compared to the other data rows?

Minor comments:

- In *"BrdU administration and immunohistochemistry*" *“scull*” should be “ *skull”.*
- Abbreviations should be defined the first time they are used in the text.

I have read this submission. I believe that I have an appropriate level of expertise to confirm that it is of an acceptable scientific standard.

# Response

**Published:** 2017-04-03

**Authors:** Gerd Kempermann

**Affiliations:** German Center for Neurodegenerative Diseases (DZNE) Dresden, Germany

1.Some details in the description of the methods are unclear. Specifically, the age of the animals used in the experiments is not clearly stated in the corresponding methods section. Although eight weeks old is mentioned in legend to figure 1 this should be clarified in the methods section because age is a strong determinant of base-line hippocampal neurogenesis.

Were animals transcardially perfused until complete elimination of blood cells before fixation? The authors should clarify this point to facilitate the reader’s interpretation of the data presented in figure 3, discarding blood cell contamination in dentate gyrus microdissected tissue samples, which could be an alternative interpretation for the differences in cytokine receptor expression in tissue vs. cultured precursor cells (last paragraph of the results section).

- Microdissection of DGs was done without prior perfusion of Nestin ^GFP^ mice (according to Ref. ^36^). Even though the dissected tissue was thoroughly washed before RNA extraction, we cannot exclude the possibility that residues of blood cells might have contributed to our quantitative RT-PCR results in whole DG samples. Still, perfusion before microdissection cannot be regarded as a guaranteed way to exclude blood cell contamination, but rather as another measure to reduce its likelihood. Apart from that, we believe that the differences in cytokine receptor expression in tissue vs. cultured precursor cells can be very well explained by the absence of virtually all bystander cells in samples of neural precursor cell (NPC) cultures.

As mentioned in the manuscript, neighbouring cells together with systemic factors and components of the extracellular matrix comprise a neurogenic niche, which provides a supportive microenvironment for the activity of the residing precursor cells whilst at the same time it regulates their expansion, differentiation and migration (Li & Xie, 2005). In this regard, several studies particularly emphasized the role of microglia as important mediators of immune-based regulatory mechanisms of adult hippocampal neurogenesis ^3,4,5^ (also: Cacci et al, 2005; Butovsky et al., 2006). The expression of several cytokine receptor molecules assessed in our study has been repeatedly demonstrated previously for cells that contribute to the neurogenic niche such as microglia, astrocytes, brain endothelial cells and mature neurons (Sawada et al., 1993; Rock et al., 2004; Mathieu et al., 2010; Szelényi, 2001). Taken together, we consider the differences in cytokine receptor expression between cultured NPCs and microdissected DG tissue to be mainly caused by the heterogeneous cellular composition of the DG and not by blood cell contamination, which rather has to be regarded as potential confounding factor, that could only be partially prevented by prior perfusion of the experimental animals.

2.  The authors suggest that one of the limitations of their study is the lack of direct characterization of the specific factor/cytokine(s) responsible for the cell-cell interaction-independent promotion of hippocampal neurogenesis they observe. However, it could have been very interesting to see some experimental effort made to support the hypothesis that the effects of Th17 cells are mediated by specific cytokines produced by these cells. This could have been done in vitro, using their cultured precursor cells, providing a stronger first proof-of-principle for the important concept they propose.

- One possible next approach is indeed to test whether cultured NPCs are in principle responsive to the candidate cytokines (TNFa, IFNg, IL-10, IL-17, IL-6). However, a possible NPC response to particular cytokines in culture will have limited value as supporting evidence of an influence of Th17 cell-secreted cytokines on hippocampal NPCs in vivo. In fact, in the brain, activated microglia are the main source of several cytokines including TNFa, IL-6, IL-10, or INFg. Activated microglia is mostly known as a suppressor, but might also act as enhancer of adult neurogenesis dependent on their phenotype or mode of activation (i.e. inflammation associated activation ^3,4,52^ versus Th cell associated IL-4 or INFg activation ^5 ^(also: Butovsky et al., 2006)). In addition some cytokines such as TNFa, IFNg, and IL-6 are expressed by astrocytes ^49^ or adult hippocampal NPCs themselves (Klassen et al., 2003). Finally, the impact of Th17 cell-derived cytokines on adult neurogenesis might be indirect, i.e. mediated by cells of the neurogenic niche, such as astrocytes, microglia or endothelial cells. In summary, Th17 influences on hippocampal neurogenesis are presumably complex and highly context-dependent, and are thus ideally addressed in vivo.

3.  In the adoptive T cell transfer experiments, control mice were injected with PBS only. Although this experimental approach provides a suitable negative control, one wonders whether the injection of other non-IL17 producing T cells (e.g. naive CD4+ differentiated into Th1 or Th2 cells) could have been a more sensible negative control for these experiments, given the specific immune functions of these different T cell sets, which are highlighted by the authors in the last sentence of their discussion. Perhaps the authors could comment on the limitations of their experimental approach in this respect?

- Our observation of an increased hippocampal precursor cell proliferation by Th17 cell-mediated indirect mechanisms provides the first evidence of a distinct CD4 ^+^ T helper subset affecting adult hippocampal neurogenesis . Certainly more work will have to be done in order to present a cohesive picture of the particular contribution of different Th cell subtypes to the pro-proliferative effect on adult NPCs. In fact, our original objective was to expand our adoptive transfer model in TCRα ^-/-^ mice towards Th1 and Th2 lineages as well. Actually, we consider the Th1 subtype to be an obvious and promising candidate. The receptor for IFN-γ, the signature cytokine of Th1 cells, is also expressed on adult neural precursor cells and several studies demonstrated that IFN-γ could influence their proliferation both and ^50,51^ (also: Butovsky et al. 2006). Moreover, Th1 cells together with Th17 cells are key factors in the pathogenesis of EAE, during which modulations of adult hippocampal neurogenesis have been observed ^9^. Unfortunately the transfer experiments involved higher effort and expenses as initially assumed. Especially the generation of naïve T cells into effector cells with a ROR-γt ^+^ IL-17 ^+^ phenotype, due to the small yields, could only be achieved by including high numbers of mice. Therefore, we could not follow up on our initial goals in the scope of the present paper.

4.  The discussion could benefit from a short paragraph including a more detailed discussion of possible pathological implications of the observations described in the paper. For example, other authors have recently found that hippocampal neurogenesis is enhanced in an arthritis model in rat (Leuchtweis *et al.*, 2014). Perhaps another interesting point of discussion could be the possible implications for depression, a disease commonly linked to alterations in hippocampal neurogenesis and possibly linked to immune function (Eyre and Baune, 2012).

- In a previous study, our group could already establish a relationship between adaptive peripheral immune responses and enhanced neurogenesis in the adult hippocampus (Wolf et al. 2009b). Similar to the work by Leuchtweis and colleagues, a T cell-dominated adaptive immune response was generated either by intraperitoneal injection of staphylococcus enterotoxin B or the induction of adjuvant-induced rheumatoid arthritis in C57BL/6 mice. Under both conditions a transient increase in hippocampal precursor cell proliferation and neurogenesis could be observed, whereas the intraperitoneal administration of lipopolysaccharide (LPS), an extremely potent activator of innate immune responses, had the opposite effect. Reduced levels of hippocampal precursor cell proliferation in different animal models of adaptive immunodeficiency and its restoration upon adoptive T cell transfers, observed in previous studies ^5,6,7^ and in the present paper, underline once again the potential role of T cell-mediated immune responses in brain homeostasis and neurogenesis.

Several immune mediators have been proposed in the pathogenesis of depression and the associated changes in adult hippocampal neurogenesis. Among them the proinflammatory cytokine IL-1β, which is known to be a key molecular mediator of innate immune responses, has repeatedly been shown to be partly responsible for the anti-neurogenic effects and depression-like behaviour in mice exposed to acute or chronic stress ^48 ^(also: Goshen et al. 2008). Regarding the opposite effects of innate versus adaptive immune responses on adult hippocampal neurogenesis, these observations could eventually be the basis for new therapeutic approaches. By steering peripheral immune responses towards an adaptive, more T cell-dominated phenotype, it might be possible to modulate the cytokine milieu in the adult brain in a way that supports neurogenesis and reduces anhedonic behaviour in patients suffering from depression.

We believe, however, that such forward-looking statements would potentially oversimplify the underlying mechanisms of CD4 ^+^ T cell-mediated neuro-immunoregulation, which still remains largely unclear. To this end, our manuscript has to be considered only as a starting point for future research. We would therefore prefer to keep the previous paragraphs out of the manuscript in order not to overstrain the current data situation.

5.  The data presented in dataset 1 seems compatible with the figures presented with the article. However, the data could be presented in a format more easily understandable for the reader. i.e. Dataset 1a is labelled “C57BL/6,TCR?+/?,TCR??/?”, while is should presumably be “C57BL/6, TCRα+/-, TCRα−/−” and the data in rows 8 and 9 seems to be in an odd format compared to the other data rows?

- It appears that there have been problems with the formatting. The datasets will therefore be converted into PDF for easier accessibility.

Minor comments: 

•   In *"BrdU administration and immunohistochemistry*" “ *scull*” should be “ *skull”.*

•   Abbreviations should be defined the first time they are used in the text.

- These points will be addressed.

# Peer review: Referee response for version 1

**DOI:** 10.5256/f1000research.4750.r5569

**Published:** 2014-07-30

**Authors:** Stefano Pluchino

**Affiliations:** 1 Department of Clinical Neurosciences, John Van Geest Centre for Brain Repair, University of Cambridge, Cambridge, UK

Study design, methods are adequate. Results are generally sound. A few claims are only in part justified by the proposed approach and controls. The importance or relevance of the myelin-specificity/reactivity in the observed promotion of endogenous hippocampal neurogenesis by adoptively transferred CD4 ^+^CD62L ^high^CD25 ^-^FoxP3 ^GFP-^ Th17 cells is not fully addressed in this paper. It is in fact quite clear that adoptive transfer of Th17 cells increases BrdU incorporation (and hence proliferation) at the level of the DG of the hippocampus. What is instead much less clear, and only speculated, is that higher proliferation leads to more neurons, and that reactivity to MOG _35-55_ (via the expression of the Vα3.2 chain of the transgenic 2D2 TCR) is indispensable for promoting adult hippocampal neurogenesis.

Main comments

1. Newly generated neurons should be quantified.
2. Experiments in Figure 2 and 3 should include also mice adoptively transferred with non-myelin reactive Th17 cells. This is a key aspect of this paper, especially in the perspective of proposing a mechanism of brain homeostasis that is promoted by a specific subset of immune cells acting in periphery (e.g. peripheral lymphoid tissues; as anticipated in the abstract). As such, also experiments in Figure 2B should include the very same positive controls as those in Figure 2A.
3. Ideally, one would like to see at least what is the contribution of the Th17 cell adoptive transfer to the cytokine milieu of the host, both at the level of the DG as well as of peripheral lymphoid tissues.

 

Minor comments

1. Wondering whether it would read better if the characterization of the pattern of expression for cytokine receptors is showed prior to the transfer experiments.
2. Nice to briefly show or comment on the clinical phenotype of that individual mouse showing remarkable accumulation of adoptively transferred Th17 cells in the brain (did it develop EAE-like signs?). 

I have read this submission. I believe that I have an appropriate level of expertise to confirm that it is of an acceptable scientific standard, however I have significant reservations, as outlined above.

# Response

**Published:** 2017-04-03

**Authors:** Gerd Kempermann

**Affiliations:** German Center for Neurodegenerative Diseases (DZNE) Dresden, Germany

1.  Newly generated neurons should be quantified.

- It is correct that we quantified neural precursor cell proliferation as one component of adult hippocampal neurogenesis. Indeed, the term neurogenesis is inclusive of the entire process from cell division to full maturity of new-born neurons after about 4 weeks (Kempermann et al., 1997). Beside proliferation, quiescence, self-renewal vs. cell fate decision (neuron vs. astrocyte, microglia; mostly few days after birth) and survival of new-born cells (30 – 70% die mainly between their first and second week) are further determinants of neurogenesis. Neurogenesis can be regarded as affected once any of the mentioned sub-processes is altered. Changes in individual sub-processes will inevitably affect the outcome, i.e. the number of new mature neurons (“net neurogenesis”), unless additional, counteracting changes occur in additional sub-processes. We, as many authors, use the term neurogenesis in this “inclusive” sense, for example in the title.

Of note, assessing new-born cells or neurons at a later stage of neurogenesis allows a better estimate of the resulting number of newly generated mature neurons (most precise at 4 weeks), but give a “collapsed” view of preceding sub-processes, i.e. assessed differences can not be assigned to a specific underlying cellular mechanism. Obviously, the reverse argumentation has to be considered as well.

 Others and we have previously shown that lack of (CD4 ^+^) T cells (depletion or deficiency) negatively impacts neurogenesis by lowering baseline proliferation and result in fewer mature neurons. Changes in further sub-processes, such as cell fate decision/differentiation (7d after division) or survival (up to 4 weeks) have not consistently been reported ^5,6,7^. In line with this, reconstitution of T-cell deficiency with (preparations that contain) CD4 ^+^ T-cells led to increased proliferation of neural precursor cells (BrdU ^+^) and/or neuronally committed cells (BrdU ^+^ after 7d and Dcx ^+^) ^5,6,7^.

 One aim of our study was to follow up on these studies and further define whether CD4 ^+^ T-cells with a specific status of activation or differentiation are responsible for the reported pro-proliferative effect. Therefore, we concentrated on the investigation of proliferation as the most validated affected measure of neurogenesis. We state this now in manuscript (Background, last sentence). We agree that this does not allow a direct conclusion on the number of newly generated neurons and we did not intend to give this impression.

 2.  Experiments in Figure 2 and 3 should include also mice adoptively transferred with non-myelin reactive Th17 cells. This is a key aspect of this paper, especially in the perspective of proposing a mechanism of brain homeostasis that is promoted by a specific subset of immune cells acting in periphery (e.g. peripheral lymphoid tissues; as anticipated in the abstract). As such, also experiments in Figure 2B should include the very same positive controls as those in Figure 2A.

- Myelin-specific T cells seem to possess a pronounced ability to promote the proliferation of neural precursor cells in the adult hippocampus ^5^. The authors of this initial report therefore suspected an underlying mechanism based on the infiltration of these self-reactive T cells into the CNS in order to directly interact with cellular components of the neurogenic niche of the dentate gyrus. In our manuscript, we report that adoptively transferred myelin-specific Th17 cells promote hippocampal cell proliferation in TCRα ^-/-^ mice evidently without entering the brain in significant numbers. Therefore, we concluded that peripheral effector functions must be responsible for this Th17 cell-mediated effect on hippocampal precursor cells. We do not, however, claim that specificity for a CNS-antigen would be a prerequisite for this effect. In fact, we do believe that Th17 cell populations with a polyclonal TCR repertoire would potentially have a similar effect on the proliferative activity of hippocampal precursor cells. We decided to use generated Th17 cells with a transgenic TCR recognizing a CNS-antigen, since we wanted to point out that under physiological conditions T cell subpopulations can affect the neurogenic region of the adult hippocampus and despite their specificity do so without infiltrating the CNS. The crucial point may simply be that the T cells become activated in the peripheral lymphoid tissues upon encounter of their cognate antigen. This should be the fact for both self-reactive as well as polyreactive T cell populations.

3. Ideally, one would like to see at least what is the contribution of the Th17 cell adoptive transfer to the cytokine milieu of the host, both at the level of the DG as well as of peripheral lymphoid tissues.

- Analyzing the cytokine milieu after adoptive T cell transfer could indeed help deciphering underlying clues of a Th17 cell-mediated regulatory mechanism of hippocampal cell proliferation. By additionally using neutralizing antibodies against certain Th17 cell-derived cytokines, one could potentially obtain even more precise results in this regard. However, the transfer experiments turned out to involve high costs and considerable expenditure of time and effort. An assay, using our cultured precursor cells, might probably be a more cost efficient alternative in the first instance (see below). We consider such experiments beyond the scope of the present manuscript.

Minor comments

 1.  Wondering whether it would read better if the characterization of the pattern of expression for cytokine receptors is showed prior to the transfer experiments.

- Our intention was to use our main finding of an increased hippocampal cell proliferation after adoptive Th17 cell transfer as a starting point and subsequently descent to the molecular level in order to find first clues for potential underlying mechanisms and provide a basis for further experimentation.

2.  Nice to briefly show or comment on the clinical phenotype of that individual mouse showing remarkable accumulation of adoptively transferred Th17 cells in the brain (did it develop EAE-like signs?).

- The affected animal did not develop clinical signs of EAE during the observation period. The cellular infiltrations found in the brain of this animal, while thwarting a quantification of hippocampal cell proliferation, were found to be limited rather than widespread, which is probably why they did not result in a clinical phenotype.
